# Interventions to control myopia progression in children: protocol for an overview of systematic reviews and meta-analyses

**DOI:** 10.1186/s13643-017-0580-x

**Published:** 2017-09-11

**Authors:** Efthymia Prousali, Asimina Mataftsi, Nikolaos Ziakas, Andreas Fontalis, Periklis Brazitikos, Anna-Bettina Haidich

**Affiliations:** 1 Aristotle University of Thessaloniki, Thessaloniki, Greece; 2 IInd Department of Ophthalmology, Aristotle University of Thessaloniki, 56403 Thessaloniki, Greece; 3 Ist Department of Ophthalmology, Aristotle University of Thessaloniki, 54621 Thessaloniki, Greece; 4 Department of Hygiene and Epidemiology, Aristotle University of Thessaloniki, 54124 Thessaloniki, Greece

**Keywords:** Overview, Myopia, Children, Spectacles, Contact lenses, Anti-muscarinic agents, Quality control

## Abstract

**Background:**

Myopia is a common visual disorder with increasing prevalence among developed countries of the world. Myopia constitutes a substantial risk factor for several ocular conditions that can lead to blindness. The purpose of this study is to conduct an overview of systematic reviews and meta-analyses in order to identify and appraise robust research evidence regarding the management of myopia progression in children and adolescents.

**Methods:**

A literature search will be conducted in MEDLINE, EMBASE, The Cochrane Database of Systematic Reviews (CDSR), Database of Abstracts of Reviews of Effects (DARE), and Health Technology Assessment (HTA) Database via Centre for Reviews and Dissemination (CRD). We will search for systematic reviews or meta-analyses that examine optical or pharmaceutical modalities for myopia control. Two independent overview authors will screen the titles and abstracts against the eligibility criteria. Individual study’s methodological quality and quality of evidence for each outcome of interest will be assessed by two independent authors using the ROBIS tool and GRADE rating, respectively. In cases of disagreement, consensus will be reached with the help of a third author. Our primary outcomes will be the mean change in refractive error, mean axial length change, and adverse events. A citation matrix will be generated, and the corrected covered area (CCA) will be estimated, in order to identify overlapping primary studies. Possible meta-biases and measures of heterogeneity will be described, and cases of dual co-authorship will be identified and discussed. If any recently published randomized controlled trials (RCTs) are detected, these will be appraised and their findings will be presented. An overall summary of outcomes will be provided using descriptive statistics and will be supplemented by narrative synthesis.

**Discussion:**

This overview will examine the high level of existing evidence for treatment of myopia progression. Efficient interventions will be identified, and side effects will be reported. The expected benefit is that all robust recent research evidence will be compiled in a single study. The results may inform future research in this area, which should provide insight into the appropriate regimes for the administration of these modalities and contribute to future guideline development.

**Systematic review registration:**

PROSPERO CRD42017068204

**Electronic supplementary material:**

The online version of this article (10.1186/s13643-017-0580-x) contains supplementary material, which is available to authorized users.

## Background

Myopia is a common visual disorder with increasing prevalence among developed countries of the world. Current evidence suggests that myopia affects a large portion of the world population, reaching over 90% in Asian countries, and typically develops in children of 6 to 8 years of age. Over the past half-century, rapid progress of myopia has been reported. Treatment of myopia at an early stage is of paramount importance, as shortsightedness poses a significant risk for several ocular disorders which could result in blindness. These conditions include retinal detachment, glaucoma, cataract, and macular degeneration [1].

A number of optical and pharmacological modalities have been widely investigated for restriction of myopia progression [2, 3]. Clinical trials on spectacles, rigid gas permeable contact lenses, progressive addition lenses, and soft contact lenses have revealed little or no long-term efficacy of these interventions in myopia control [4, 5]. Both tropicamide combined with bifocals and timolol have also failed to show a significant effect on slowing myopia progression [6, 7]. Two randomized controlled trials on pirenzepine revealed encouraging findings; however, research has not been continued for this agent [8–10]. Contradictory evidence exists regarding the effect of undercorrection on myopia progression. Two randomized controlled trials reported that undercorrection enhances myopia development [11, 12]. Recent evidence shows that undercorrection restricts myopia progression compared to full correction in already myopic children, which is in line with former findings from animal studies [13]. Acupuncture has also been investigated for myopia control, but insufficient evidence exists regarding its appropriateness for clinical use [14].

Recent studies have reported positive findings for atropine eye drops and orthokeratology in the management of juvenile myopia [15–18]. In addition, bifocal and multifocal soft contact lenses designed with new technology constitute an emerging treatment with promising results [19]. Increased outdoor exposure has also been described to have a protective effect on myopia development [20].

Currently, there are no generally accepted guidelines on the treatment of myopic progression. Various refractive and pharmaceutical interventions have been investigated, but atropine which appears the most beneficial agent has not been approved by the Food and Drug Administration (FDA). Associated adverse events have prevented efficient interventions from becoming widely adopted for myopia treatment [21, 22]. Myopia causes considerable medical and economic impact on society. The cost of treatment is significant to both individuals and society. Annual expenses for myopia treatment are estimated to be greater than for other ocular pathologies, as well as for non-ocular chronic conditions. The quality of life of individuals is also affected due to functional, cosmetic, and psychological implications [23–25].

Further investigation is warranted, due to the existing increasing prevalence of myopia in the worldwide population [26]. Existing evidence, although at a high level, has failed to convince ophthalmologists to uniformly embrace treatments for myopia progression control. A study design that has recently gained interest is the overview of systematic reviews or umbrella review that attempts to bring together and treat synthetically the evidence from systematic reviews with or without meta-analyses in a given domain [27–29]. Thus, there is no overview in existing literature synthesizing the information provided by systematic reviews and meta-analyses on slowing myopia progression in children, and this is the aim of the present study.

## Methods

### Protocol and registration

This overview has been registered in the PROSPERO database (CRD42017068204) and has been prepared in consultation with the PRISMA-P statement [30, 31]. PRISMA-P checklist is provided as Additional file 1. Any amendments to the protocol until completion of the overview shall be provided with reasons and shall be available to public view.

### Information sources and search strategy

A purposive literature search will be conducted in the Cochrane Database of Systematic Reviews (CDSR), Database of Abstracts of Reviews of Effects (DARE), and Health Technology Assessment (HTA) Database via Centre for Reviews and Dissemination (CRD) using the keyword “myopia.” A more comprehensive search strategy will be applied in MEDLINE and EMBASE, using medical subject headings (MeSH) and text words related to spectacles, contact lenses, anti-muscarinic agents, myopia, and children [4, 32]. Database search date is 15 January 2017. For all included studies, reference lists will be also searched. MEDLINE search strategy and keywords are provided in Additional file 2. No language, study type, or date restrictions will be used.

### Research question

What is the efficacy and safety of optical and pharmacological interventions used to control myopia progression in children and adolescents?

### Eligibility criteria

The overview question being addressed is best described by the following PICOS (participants, intervention, comparator, outcomes, study design) format.

#### Participants

Our overview targets are children and adolescents, equal to or less than 18 years of age (at baseline), diagnosed with myopia defined as spherical equivalent refraction ≤ − 0.25 diopters, with or without astigmatism, and without any ocular comorbidities including strabismus and amblyopia. Animals, adult population, patients not suffering from myopia, or patients with myopia and strabismus/amblyopia will be excluded. Studies related to surgical interventions for myopia correction, e.g., refractive surgery will not be considered.

#### Interventions

Any optical or pharmacological intervention for controlling myopia progression will be identified. No restriction on duration and dose (if applicable) of treatment will be imposed.

#### Comparators

Comparators will be the use of single-vision spectacles, contact lenses, (sham) acupuncture, or placebo for controlling myopia progression. No restriction on duration of treatment will be imposed.

#### Outcomes

Our primary outcomes will regard myopia progression and axial elongation as efficacy criteria. Myopia progression will be assessed as mean change in refractive error, measured in diopters, per year. Mean change in axial length, measured in millimeters, per year, will also be evaluated. Outcomes reporting a change in other than a 12-month period will also be accepted and described. Reported adverse events will be regarded as safety criteria. A descriptive approach of side effects will be performed, and odd ratios will be presented, if provided by the authors.

#### Study design

We will consider systematic reviews or meta-analyses of randomized controlled trials (RCTs), pseudo-RCTs, and cohort and case-control studies. Network meta-analyses, if available, will also be reviewed. In order to ensure literature saturation, we aim to consider any recently published RCTs found to not have been included in the meta-analyses. Should recent RCTs be identified, these will also be appraised and their findings will be presented and discussed. No language limitation will be imposed. Only human studies with full text available will be analyzed.

Systematic reviews or meta-analyses of poor quality cohort studies, case series, case reports, or expert opinions will not be considered. Narrative reviews that do not systematically search the literature and do not critically appraise the quality of included studies will be excluded.

### Data management and extraction

Two independent authors will perform all screening steps. Title and abstract screening will be conducted with the Mendeley citation management software. The overview authors will screen the titles and abstracts against the eligibility criteria and obtain full reports for all titles that appear to meet the inclusion criteria, or where there is uncertainty. One team of two independent overview authors will manage data in duplicate from each eligible study, using a data collection form in Microsoft Excel, designed specifically to include all the data required. We will only extract data of the included systematic reviews or meta-analyses and not directly from the primary studies, as this process would be beyond the scope of our overview. In cases of disagreement, consensus will be reached with the help of a third author.

For each included study, information will be extracted on the type of included study (systematic review, systematic review and meta-analysis, meta-analysis, or network meta-analysis); publication date; number of databases sourced and searched; last literature search; type of included primary studies (randomized controlled trials, observational studies, or both); country/countries of origin of studies included in each review; number of primary studies in each review; source of financial support (if any); participants’ characteristics (age range); total sample size (or range of sizes of primary studies); type of treatment (optical or pharmaceutical) and dose when applicable; type of control (optical, pharmaceutical, or placebo) and dose when applicable; follow-up period; outcomes that are relevant to our overview question; results; major conclusions; instrument used to appraise the primary studies and the rating of their quality; metric used and effect size (for meta-analyses); *p*-value (for meta-analyses); confidence intervals (for meta-analyses); additional analyses (e.g. subgroup analysis, meta-regression, sensitivity analysis, for meta-analyses); and handling of heterogeneity (fixed/random effects model, funnel plot, for meta-analyses).

### Risk-of-bias assessment

Two overview authors will independently assess the methodological quality of the included systematic reviews and meta-analyses using the ROBIS tool. A qualitative, domain-based assessment will be performed with ROBIS, evaluating eligibility criteria, identification and selection of primary studies, data collection and study appraisal, and synthesis and findings. A tabular presentation of ROBIS assessment for each included review will be provided and will enable comparisons between studies and detection of the possible presence of bias [33]. We will also provide a table presenting a risk-of-bias assessment of primary studies and relevant tool utilized by each eligible review. If the risk of bias of primary studies has not been evaluated by one or more reviews, we will appraise these studies and present our assessment in a relevant table. The strength of evidence for each outcome of interest across the eligible systematic reviews will be evaluated by two independent authors using the GRADE rating [34–36]. In order to minimize the subjectivity of quality assessment process, a third reviewer will be involved to resolve any discrepancies.

The list of included primary studies will be reviewed in order to identify overlapping studies considered in two or more eligible reviews. We will generate a citation matrix presenting all the included reviews and primary publications. We will estimate the overlap by calculating the corrected covered area (CCA), to assess if specific primary studies are overrepresented. CCA will reflect the degree of actual overlap, as it is not influenced by large reviews. Should high or very high overlap be detected, we will retain the review which will be (1) the most recent, (2) containing a higher amount of information, and (3) the most rigorous in terms of methodology, as assessed by ROBIS tool and GRADE scale. We will examine and record whether the results of overlapping studies are concordant, and if many relevant primary studies would be excluded as a result of the above approach [37].

Two independent overview authors will also examine possible presence of meta-biases, including publication bias, selective outcome reporting, and dual co-authorship. Handling of heterogeneity for meta-analyses and other potential sources of bias will be described [38–41].

### Data synthesis

To summarize findings, a descriptive synthesis will be performed. Specifically, we will provide tables that include summaries of study characteristics, quality assessment, and major conclusions. Methodological rigor and quality of evidence of the included studies will be reflected by ROBIS and GRADE assessments. The extent of overlapping primary studies will be displayed in our citation matrix, and the corrected covered area (CCA) will provide an estimate of the actual overlap. Our overview outcomes regarding the efficacy and safety of myopia treatments will be stratified and presented according to the type of intervention. For studies examining the same interventions, we will state whether the reported conclusions are concordant. Should any recent randomized controlled trials be found to not have been included in the meta-analyses, these will also be appraised and their findings will be presented and discussed.

## Discussion

Currently, no consensus has been reached regarding the management of myopic progression and no universal guidelines have been established. This study aims to qualitatively synthesize systematic reviews and meta-analyses in order to identify and appraise high-level research evidence regarding myopia control in children and adolescents. Efficient interventions will be identified and side effects will be reported. Recently published randomized controlled trials will also be presented to provide an insight into the most recent available evidence for retarding myopia progression. The results may guide future research in this area and contribute to guideline development.

## Additional files

Additional file 1: PRISMA-P checklist. (DOCX 36 kb)

Additional file 2: MEDLINE search strategy. (DOCX 16 kb)

## Abbreviations

CCA: Corrected covered area
CDSR: Cochrane Database of Systematic Reviews
CRD: Centre for Reviews and Dissemination
DARE: Database of Abstracts of Reviews of Effects
FDA: Food and Drug Administration
HTA: Health Technology Assessment
PICOS: Population Intervention Comparison Outcome(s) Study design
PRISMA: Preferred Reporting Items for Systematic Reviews and Meta-analyses
PROSPERO: International prospective register of systematic reviews
RCTs: Randomized controlled trials
ROBIS: Risk of Bias in Systematic Reviews

## References

[1] Leo S-W, Young TL (2011). An evidence-based update on myopia and interventions to retard its progression. J AAPOS.

[2] Walline JJ (2016). Myopia Control: a review. Eye Contact Lens.

[3] Aller TA (2014). Clinical management of progressive myopia. Eye (Lond).

[4] Walline JJ, Lindsley K, Vedula SS, Cotter SA, Mutti DO, Twelker JD. Interventions to slow progression of myopia in children. Cochrane Database Syst Rev. 2011;12:CD004916. doi:10.1002/14651858.CD004916.pub3. 10.1002/14651858.CD004916.pub3 PMC4270373 22161388

[5] Saw SM, Shih-Yen EC, Koh A, Tan D (2002). Interventions to retard myopia progression in children: an evidence-based update. Ophthalmology.

[6] Schwartz JT. Results of a monozygotic cotwin control study on a treatment for myopia. Prog Clin Biol Res. 1981;69. 7031687

[7] Jensen H. Myopia progression in young school children. A prospective study of myopia progression and the effect of a trial with bifocal lenses and beta blocker eye drops. Acta Ophthalmol Suppl (Oxf). 1991:1–79. 1663308

[8] Tan DTH, Lam DS, Chua WH, Shu-Ping DF, Crockett RS, Group APS (2005). One-year multicenter, double-masked, placebo-controlled, parallel safety and efficacy study of 2% pirenzepine ophthalmic gel in children with myopia. Ophthalmology.

[9] Siatkowski RM, Cotter SA, Crockett RS, Miller JM, Novack GD, Zadnik K (2008). Two-year multicenter, randomized, double-masked, placebo-controlled, parallel safety and efficacy study of 2% pirenzepine ophthalmic gel in children with myopia. J AAPOS.

[10] Galvis V, Tello A, Parra MM, Rodriguez CJ, Blanco O, V. G, et al. Re: Chia et al.: Five-year clinical trial on atropine for the treatment of myopia 2: myopia control with atropine 0.01% eyedrops (Ophthalmology 2016;123:391–9). Ophthalmology. 2016;123:391–399. 10.1016/j.ophtha.2015.12.037 27210609

[11] Chung K, Mohidin N, O’Leary DJ. Undercorrection of myopia enhances rather than inhibits myopia progression. Vis Res. 2002;42:2555–9. doi:10.1016/S0042-6989(02)00258-4. 10.1016/s0042-6989(02)00258-4 12445849

[12] Adler D, Millodot M. The possible effect of undercorrection on myopic progression in children. Clin Exp Optom. 2006;89:315–21. doi:10.1111/j.1444-0938.2006.00055.x. 10.1111/j.1444-0938.2006.00055.x 16907670

[13] Sun YY, Li SM, Li SY, Kang MT, Liu LR, Meng B, et al. Effect of uncorrection versus full correction on myopia progression in 12-year-old children. Graefes Arch Clin Exp Ophthalmol. 2016:1–7. doi:10.1007/s00417-016-3529-1. 10.1007/s00417-016-3529-1 27796670

[14] Wei ML, Liu JP, Li N, Liu M (2011). Acupuncture for slowing the progression of myopia in children and adolescents. Cochrane Database Syst Rev.

[15] Shih KC, Chan TC-Y, Ng AL-K, Lai JS-M, Li WW-T, Cheng AC-K (2016). Use of atropine for prevention of childhood myopia progression in clinical practice. Eye Contact Lens.

[16] Si J-K, Tang K, Bi H-S, Guo D-D, Guo J-G, Wang X-R (2015). Orthokeratology for myopia control: a meta-analysis. Optom Vis Sci.

[17] Smith MJ, Walline JJ (2015). Controlling myopia progression in children and adolescents. Adolesc Health Med Ther.

[18] Liu YM, Xie P (2016). The safety of orthokeratology—a systematic review. Eye Contact Lens.

[19] Li SM, Kang MT, Wu SS, Meng B, Sun YY, Wei SF, et al. Studies using concentric ring bifocal and peripheral add multifocal contact lenses to slow myopia progression in school-aged children: a meta-analysis. Ophthalmic Physiol Opt. 2017;37:51–9. doi:10.1111/opo.12332. 10.1111/opo.12332 27880992

[20] Sherwin JC, Reacher MH, Keogh RH, Khawaja AP, Mackey DA, Foster PJ (2012). The association between time spent outdoors and myopia in children and adolescents: a systematic review and meta-analysis. Ophthalmology.

[21] Iribarren R, Morgan I (2017). Myopia - EyeWiki.

[22] Wu P-C, Huang H-M, Yu H-J, Fang P-C, Chen C-T. Epidemiology of Myopia. Asia-Pacific J Ophthalmol (Philadelphia, Pa). 2016;5:386–93. doi:10.1097/APO.0000000000000236. 10.1097/APO.0000000000000236 27898441

[23] Foster PJ, Jiang Y (2014). Epidemiology of myopia. Eye (Lond).

[24] Fricke T, Holden B, Wilson D, Schlenther G, Naidoo K, Resnikoff S (2012). Global cost of correcting vision impairment from uncorrected refractive error. Bull World Health Organ.

[25] Zheng YF, Pan CW, Chay J, Wong TY, Finkelstein E, Saw SM. The economic cost of myopia in adults aged over 40 years in Singapore. Investig Ophthalmol Vis Sci. 2013;54:7532–7. doi:10.1167/iovs.13-12795. 10.1167/iovs.13-12795 24159089

[26] Morgan IG (2016). What public policies should be developed to deal with the epidemic of myopia?. Optom Vis Sci.

[27] Becker LA, Oxman AD (2011). Chapter 22: overviews of reviews. In: Higgins JPT, Green S, editors. Cochrane handbook for systematic reviews of interventions (version 5.1.0). The Cochrane Collaboration.

[28] Lavis JN (2009). How can we support the use of systematic reviews in policymaking?. PLoS Med.

[29] Thomson D, Russell K, Becker L, Klassen T, Hartling L (2010). The evolution of a new publication type: steps and challenges of producing overviews of reviews. Res Synth Methods.

[30] Moher D, Shamseer L, Clarke M, Ghersi D, Liberati A, Petticrew M (2015). Preferred reporting items for systematic review and meta-analysis protocols (PRISMA-P) 2015 statement. Syst Rev.

[31] Shamseer L, Moher D, Clarke M, Ghersi D, Liberati A, Petticrew M, et al. Preferred reporting items for systematic review and meta-analysis protocols (PRISMA-P) 2015: elaboration and explanation. BMJ. 2015;349:g7647. doi:10.1136/bmj.g7647. Accessed 18 May 2017. 10.1136/bmj.g7647 25555855

[32] Boluyt N, Tjosvold L, Lefebvre C, Klassen TP, Offringa M (2008). Usefulness of systematic review search strategies in finding child health systematic reviews in MEDLINE. Arch Pediatr Adolesc Med.

[33] Whiting P, Savović J, Higgins JPT, Caldwell DM, Reeves BC, Shea B (2016). ROBIS: a new tool to assess risk of bias in systematic reviews was developed. J Clin Epidemiol.

[34] Balshem H, Helfand M, Schünemann HJ, Oxman AD, Kunz R, Brozek J (2011). GRADE guidelines: 3. Rating the quality of evidence. J Clin Epidemiol.

[35] Guyatt GH, Oxman AD, Kunz R, Vist GE, Falck-Ytter Y, Schünemann HJ (2009). GRADE: what is “Quality of evidence” and why is it important to clinicians?. Chinese J Evidence-Based Med.

[36] Guyatt G, Oxman AD, Sultan S, Brozek J, Glasziou P, Alonso-Coello P (2013). GRADE guidelines: 11. Making an overall rating of confidence in effect estimates for a single outcome and for all outcomes. J Clin Epidemiol.

[37] Ballard M, Montgomery P. Risk of bias in overviews of reviews: a scoping review of methodological guidance and four-item checklist. Res Synth Methods. 2017; doi:10.1002/jrsm.1229. 10.1002/jrsm.1229 28074553

[38] Thornton A, Lee P (2000). Publication bias in meta-analysis: Its causes and consequences. J Clin Epidemiol.

[39] Aromataris E, Fernandez R, Godfrey C, Holly C, Tungpunkom P (2014). Methodology for JBI umbrella reviews. Joanna Briggs Inst Rev Man.

[40] Higgins J, Green S (2008). Cochrane Handbook for Systematic Cochrane Handbook for Systematic Reviews of.

[41] Büchter RB, Pieper D (2016). Most overviews of Cochrane reviews neglected potential biases from dual authorship. J Clin Epidemiol.

