# Supplementary material for: Interventions to control myopia progression in children: protocol for an overview of systematic reviews and meta-analyses
Source: Syst Rev. 2017 Sep 11;6:188. doi: 10.1186/s13643-017-0580-x (PMC5594593; doi:10.1186/s13643-017-0580-x)
Supplement: Supplementary file 2 — MEDLINE search strategy. (DOCX 16 kb) [file 13643_2017_580_MOESM2_ESM.docx]

**Additional file 2: MEDLINE Search Strategy**

The search on MEDLINE and EMBASE electronic databases was performed on the 15^th^ January 2017 using the NICE Healthcare Databases Advanced Search (HDAS) interface.

MEDLINE Search Strategy:

1. exp MYOPIA/
2. (myop*).ti,ab
3. (shortADJ3sight*).ti,ab
4. (1 OR 2 OR 3)
5. exp EYEGLASSES/
6. (spectacles OR glasses).ti,ab
7. exp “CONTACT LENSES”/
8. (contactADJ2lens*).ti,ab
9. exp “MUSCARINIC ANTAGONISTS”/
10. (muscarinicADJ2antagonist*).ti,ab
11. (antiADJ1muscarinic).ti,ab
12. exp “CHOLINERGIC ANTAGONISTS”/
13. (cholinergicADJ2antagonist*).ti,ab
14. (antiADJ1cholinergic).ti,ab
15. exp ATROPINE/
16. (atropine*).ti,ab
17. exp CYCLOPENTOLATE/
18. (cyclopentolate*).ti,ab
19. exp PIRENZEPINE/
20. (pirenzepine*).ti,ab
21. exp TROPICAMIDE/
22. (tropicamide*).ti,ab
23. exp TIMOLOL/
24. (timolol*).ti,ab
25. exp PHENYLEPHRINE/
26. (phenylephrine*).ti,ab
27. (5 OR 6 OR 7 OR 8 OR 9 OR 10 OR 11 OR 12 OR 13 OR 14 OR 15 OR 16 OR 17 OR 18 OR 19 OR 20 OR 21 OR 22 OR 23 OR 24 OR 25 OR 26)
28. (4 AND 27)
29. exp INFANT/
30. (infant*).ti,ab
31. (infancy).ti,ab
32. (newborn*).ti,ab
33. (baby*).ti,ab
34. (babies).ti,ab
35. (neonat*).ti,ab
36. (preterm*).ti,ab
37. (prematur*).ti,ab
38. (postmatur*).ti,ab
39. exp CHILD/
40. (child*).ti,ab
41. (schoolchild*).ti,ab
42. (school age*).ti,ab
43. (preschool*).ti,ab
44. (kid*).ti,ab
45. (toddler*).ti,ab
46. exp ADOLESCENT/
47. (adoles*).ti,ab
48. (teen*).ti,ab
49. (boy*).ti,ab
50. (girl*).ti,ab
51. exp MINORS/
52. (minors*).ti,ab
53. exp PUBERTY/
54. (pubert*).ti,ab
55. (pubescen*).ti,ab
56. (prepubscen*).ti,ab
57. exp PEDIATRICS/
58. (pediatric*).ti,ab
59. (paediatric*).ti,ab
60. (peadiatric*).ti,ab
61. exp SCHOOLS/
62. (nursery school*).ti,ab
63. (kindergar*).ti,ab
64. (primary school*).ti,ab
65. (secondary school*).ti,ab
66. (elementary school*).ti,ab
67. (high school*).ti,ab
68. (highschool*).ti,ab
69. (29 OR 30 OR 31 OR 32 OR 33 OR 34 OR 35 OR 36 OR 37 OR 38 OR 39 OR 40 OR 41 OR 42 OR 43 OR 44 OR 45 OR 46 OR 47 OR 48 OR 49 OR 50 OR 51 OR 52 OR 53 OR 54 OR 55 OR 56 OR 57 OR 58 OR 59 OR 60 OR 61 OR 62 OR 63 OR 64 OR 65 OR 66 OR 67 OR 68)
70. (28 AND 69)
